# Hypogonadism and Cryptorchidism

**DOI:** 10.3389/fendo.2019.00906

**Published:** 2020-01-15

**Authors:** Wiwat Rodprasert, Helena E. Virtanen, Juho-Antti Mäkelä, Jorma Toppari

**Affiliations:** ^1^Research Centre for Integrative Physiology and Pharmacology, Institute of Biomedicine, University of Turku, Turku, Finland; ^2^The Population Research Centre, University of Turku, Turku, Finland; ^3^Department of Pediatrics, Turku University Hospital, Turku, Finland

**Keywords:** undescended testis, testosterone, gonadotropins, Leydig cell, Sertoli cell

## Abstract

Congenital cryptorchidism (undescended testis) is one of the most common congenital urogenital malformations in boys. Prevalence of cryptorchidism at birth among boys born with normal birth weight ranges from 1.8 to 8.4%. Cryptorchidism is associated with a risk of low semen quality and an increased risk of testicular germ cell tumors. Testicular hormones, androgens and insulin-like peptide 3 (INSL3), have an essential role in the process of testicular descent from intra-abdominal position into the scrotum in fetal life. This explains the increased prevalence of cryptorchidism among boys with diseases or syndromes associated with congenitally decreased secretion or action of androgens, such as patients with congenital hypogonadism and partial androgen insensitivity syndrome. There is evidence to support that cryptorchidism is associated with decreased testicular hormone production later in life. It has been shown that cryptorchidism impairs long-term Sertoli cell function, but may also affect Leydig cells. Germ cell loss taking place in the cryptorchid testis is proportional to the duration of the condition, and therefore early orchiopexy to bring the testis into the scrotum is the standard treatment. However, the evidence for benefits of early orchiopexy for testicular endocrine function is controversial. The hormonal treatments using human chorionic gonadotropin (hCG) or gonadotropin-releasing hormone (GnRH) to induce testicular descent have low success rates, and therefore they are not recommended by the current guidelines for management of cryptorchidism. However, more research is needed to assess the effects of hormonal treatments during infancy on future male reproductive health.

## Introduction

Cryptorchidism (undescended testis, maldescendus testis) is a condition in which one or both testes fail to descend into the bottom of the scrotum (1). Instead, the testis is found at a location along the normal route of testicular descent, and it may have an intra-abdominal, inguinal, suprascrotal, or high scrotal position. Congenital cryptorchidism is one of the most common congenital malformations in boys. Its prevalence at birth among boys with birth weight more than 2,500 g has been reported to range from 1.8 to 8.4% (2). The prevalence at the age of 3 months and 1 year is 0.9–1.6 and 1.0–1.5%, respectively, which are lower than the prevalence at birth due to spontaneous testicular descent (2, 3). Prevalence at birth for boys with preterm birth and/or low birth weight varies from 1.1 to 45.3% (3).

Cryptorchidism is associated with a future risk of poor semen quality and increased incidence of testicular germ cell tumors (TGCT) (4, 5). The risk of TGCT is increased 2–5-fold when compared to the general population (5, 6). Notably, early treatment of cryptorchidism does not significantly reduce the risk of TGCT later in life (7, 8). These findings support the testicular dysgenesis syndrome (TDS) hypothesis, which suggests that cryptorchidism and TGCT have a common origin in fetal life (9, 10).

There is evidence to support that cryptorchidism is associated with decreased testicular hormone concentrations. On the other hand, congenital hypogonadism may have cryptorchidism as one of the manifestations. The classical definition of hypogonadism is testicular dysfunction associated with androgen deficiency (11). However, Rey et al. have proposed a more comprehensive description, which would include age-dependent testicular dysfunction, including impaired Leydig cell and/or Sertoli cell function and/or a disorder of spermatogenesis (11).

In this article, we will review the literature from human studies focusing on the associations between congenital cryptorchidism and reproductive hormone levels. We conducted a Pubmed search between March and October 2019 with no publication date limit. We used the following keywords for literature search: “cryptorchidism,” “undescended testis,” “hypogonadism,” “hormone,” “testicular descent,” “androgen,” “testosterone,” “FSH,” “follicle-stimulating hormone,” “LH,” “luteinizing hormone,” “inhibin,” “INSL3,” “insulin-like peptide 3,” “Sertoli cell,” “Leydig cell,” “puberty,” “cord blood,” “mini-puberty,” “prepuberty,” “adult,” “fertility,” “anti-Müllerian hormone,” “reproductive,” “gonadotropin,” “syndromic cryptorchidism,” “acquired cryptorchidism,” “hypogonadotropic hypogonadism,” “congenital hypogonadotropic hypogonadism,” “hCG,” “human chorionic gonadotropin,” “LHRH,” “GnRH,” “post-natal,” “hormonal therapy,” “orchiopexy,” “fertility,” “infertility,” “testicular dysgenesis syndrome,” “Klinefelter syndrome,” and “Kallmann syndrome.” We limited the literature search to articles in English. Full texts of the relevant articles were obtained. Reference lists of these articles were also checked to identify additional studies. We classified the studies according to different periods of life—birth, mini-puberty, prepuberty, puberty, and adulthood. The connection of cryptorchidism with semen quality and/or fertility have been extensively reviewed elsewhere (12, 13), therefore these topics are not the focus of this article.

## Testicular Location

**Figure 1:**
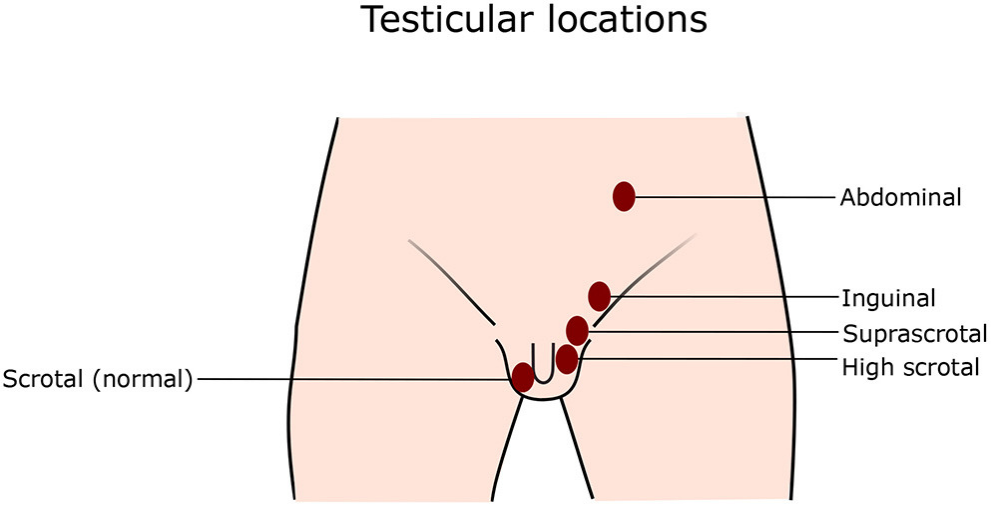
Testicular locations. Normally, both testes locate at the bottom of the scrotum. In cryptorchidism, one or both testes do not stay at the normal position, but anywhere along the normal path of testicular descent as illustrated in the figure.

Normal position of the testis is inside the scrotum, where the center of the testis is at or below the border between the upper and lower half of the scrotum (14). *Retractile testis* is a testis that can be pulled to the bottom of the scrotum and does not return up immediately after release. A *High scrotal position* means that a testis is at the upper part of the scrotum. Some testes at the high scrotal position can be drawn to the middle or the bottom of the scrotum, but immediately after release they return to the original position, which distinguishes them from retractile testes. *Suprascrotal position* is the area above the scrotum. The testis may also be close to the external inguinal ring. The testis at the *inguinal position* stays inside the inguinal canal, which is sometimes difficult to palpate, but can be visible with ultrasonography (14). Retractile testes and testes with scrotal position are considered normal. In rare cases, the testis is located outside the normal path of the testicular descent, and is called *ectopic testis*. In this case, the testis can be located at, for instance, perineal, femoral, or pubopenile area, or at a crossed scrotal position. When a testis is non-palpable, it probably has an inguinal, abdominal, or ectopic location, or it has vanished (14–16). See Figure 1 for the illustration of different testicular positions.

## Physiology of Testicular Hormone Production

Fetal Leydig cells produce testosterone and insulin-like peptide 3 (INSL3). According to the literature they start to secrete androgens during gestational weeks (GW) 8–10, and the highest levels of these hormones are measured around GW 16 (17–20). Dihydrotestosterone (DHT) converted from testosterone by 5-alpha reductase mostly in peripheral tissues, is crucial for the differentiation of male external genitalia and prostate during GW 8–12 (17–19). Androgen secretion in fetal Leydig cells is stimulated by human chorionic gonadotropin (hCG) from the placenta during the first trimester of pregnancy and later by luteinizing hormone (LH) from the anterior pituitary gland of the developing fetus (19, 21). Placental hCG also regulates the secretion of INSL3, which is a peptide hormone of insulin-related gene family (22), and essential for the transabdominal phase of testicular descent (23). INSL3 is also involved in testicular descent in mice by acting on the gubernaculum by binding to relaxin-family peptide receptor 2 (RXFP2), which is also known as leucine-rich repeat-containing G protein-coupled receptor 8 (LGR8) and G protein-coupled receptor affecting testis descent (GREAT) (24, 25).

Plasma hCG levels drop rapidly after GW 10 and continue to decline slowly after GW 20 (21). Pituitary gonadotropes start producing FSH and LH around GW 9, and the hormones are detected in the fetal blood by GW 12–14 (26). The gonadotropin levels reach their peaks at midgestation and subsequently decrease toward the time of delivery (21, 26). LH shares the same receptor (LHCGR) with hCG on the Leydig cells (21). LH takes over the role of hCG to control androgen and INSL3 secretion from Leydig cells at around GW 15–20 (21, 27). FSH has an essential role in promoting Sertoli cell proliferation and stimulation of anti-Müllerian hormone (AMH) and inhibin B production from Sertoli cells (28–30). The gonadotropin levels decline steadily toward the time of delivery (31). For a comprehensive overview of reproductive hormone production during pregnancy, see Scott et al. (32).

Gonadotropin and testosterone levels start to rise again about 1 week after birth and peak at the age of 1–3 months, a period also known as mini-puberty (30, 31). During this period, there is an increased proliferation of Leydig cells (33), Sertoli cells (34), and germ cells (35). The post-natal surge of gonadotropins is also associated with accelerated penile growth (36), testicular descent (37), increased prostatic activity (38), and male sex-typed behavior at 14 months (39). Mini-puberty is also associated with an increase in testicular volume and inhibin B levels, reflecting the growing number of Sertoli cells (40–42). Furthermore, it has also been shown that the transformation of gonocytes into type A-dark (Ad) spermatogonia, i.e., stem cells for spermatogenesis, is completed during this period (43, 44), and therefore this developmental period is essential for adult fertility (44). Interestingly, the testosterone surge stimulated by increased gonadotropin levels during mini-puberty has also been suggested to have a role in this process as one study showed that cryptorchid boys who received hCG treatment before orchiopexy ended up having a higher number of Ad spermatogonia than boys who underwent the operation without receiving hCG (44). However, the role of androgen in this process is still unclear, and animal studies do not provide a strong support for it (45). Notably, however, the timing of germ cell development is different between mouse and man, and the results from mouse work cannot be directly extrapolated to human (46). After 6–9 months of age, LH and testosterone levels decrease rapidly to very low or undetectable levels, while the decline in FSH level is also substantial but remains detectable. Plasma levels for all these hormones remain low for the rest of the childhood and until the time of puberty, which is characterized by reactivation of the hypothalamic-pituitary-testicular (HPT) axis, and its sustained function continuing throughout adulthood (47, 48).

Mini-puberty is also associated with elevated levels of inhibin B, INSL3, and AMH (49, 50). The inhibin B level at 3 months of age is even higher than in adulthood. After 15 months of age, the inhibin B level decreases, however it is still detectable throughout childhood (30, 47, 48, 51, 52). AMH level is still high after the age of 6 months, reflecting the functional immaturity of Sertoli cells (53). Interestingly, its function in postnatal males is still unclear. Both inhibin B and AMH [also known as Müllerian inhibiting substance (MIS)] are produced by Sertoli cells. AMH is a glycoprotein homodimer, which is a member of the transforming growth factor-β (TGF-β) superfamily, along with inhibins, activins, bone morphogenetic proteins (BMPs), and growth differentiation factors (GDFs) (54, 55). Sertoli cells start secreting AMH from approximately GW 8–9, which causes regression of Müllerian duct-derived structures, i.e., fallopian tubes, uterus and upper vagina (20, 56, 57). AMH secretion continues until the start of puberty, demarcating functional maturation of Sertoli cells (56, 57). Inhibin B is a heterodimeric glycoprotein hormone in the TGF-β superfamily. It is composed of one inhibin α subunit and one inhibin β_B_ subunit. Inhibin B suppresses FSH secretion from the anterior pituitary gland (51). Interestingly, in childhood inhibin B is secreted solely by Sertoli cells. However, in the adult testis, mature Sertoli cells produce only α-subunit and β_B_-subunit is produced by germ cells during early spermatogenesis (51, 58).

AMH is a testis-specific marker because an extragonadal source of AMH production is not found (29). It is detected at all ages in males (59). FSH is a moderate inducer of AMH production, whereas testosterone is a potent inhibitor of its synthesis (29). AMH level is high in the fetus, remains rather stable until birth, and is elevated during mini-puberty (60, 61) due to FSH surge. Although testosterone level is also high at this time, lack of androgen receptor (AR) expression renders Sertoli cells unresponsive to the inhibitory effect of testosterone (62). During prepuberty, Sertoli cells continue producing high levels of AMH, even without FSH stimulation, because there is no strong inhibitory effect on AMH production by testosterone which is nearly absent (62). Sertoli cells start to express AR in the prepubertal testis (62). When boys enter puberty, the increased testosterone production causes high intratesticular testosterone levels, which strongly inhibit AMH production and overcome the stimulatory effect of FSH (55), hence resulting in a low AMH level. This effect persists until adulthood (62). Therefore, serum AMH is a biomarker of immature Sertoli cell function during prepuberty (62). In humans, the presence of androgen receptors in Sertoli cells is necessary for the downregulation of AMH expression (63). However, the downregulation of AMH expression in mouse Sertoli cells does not require androgen action (64). Mice with a selective ablation of androgen receptor in Sertoli cells (SCARKO mice) or androgen receptor knockout (ARKO) mice with an ubiquitous ablation of androgen receptor display a strong downregulation of AMH expression during the first weeks after birth and early puberty (64). Notably, in men with congenital hypogonadotropic hypogonadism (CHH), AMH levels remain high after puberty due to the lack of testosterone effect (65).

In adult men, the hypothalamus secretes GnRH in a pulsatile manner stimulating the pituitary gonadotrophs to release FSH and LH. FSH stimulates the secretion of inhibin B from Sertoli cells (66). Both FSH and high intratesticular testosterone promote spermatogenesis. LH acts on Leydig cells in the testicular interstitium and stimulates the secretion of INSL3 and testosterone. INSL3, unlike testosterone, is not prone to the acute fluctuations of the HPG axis activity (67) because it is dependent on the long-term trophic effect of LH (68), and its levels thus reflect the number of Leydig cells and their differentiation status (67).

FSH and inhibin B are recognized as markers of Sertoli cell function and spermatogenesis (66, 69, 70). The correlation between FSH and inhibin B levels varies according to age, suggesting that the canonical negative feedback regulation does not apply at every stage of development. The relationship between serum FSH and inhibin B levels during infancy is not clear, but the negative association is likely established already at this age (40, 51, 71). However, during early puberty, a positive association between FSH and inhibin B levels is found. In contrast, a negative association is observed during late puberty (72), and it continues into adulthood (69). A negative correlation is also observed in men with a childhood history of cryptorchidism (73).

## Physiology of Testicular Descent

Testicular descent in male fetuses is a complex process. Normal function of the HPT axis and androgen secretion and action are crucial for normal testicular descent (74). The physiology of testicular descent has been extensively reviewed elsewhere (23, 46, 75). Here, we describe this process only briefly.

Bipotential gonads are formed during weeks 4–6 of embryonic development (76). They are located on the dorsal wall of the body cavity. The primordial germ cells migrate from the extraembryonic mesoderm to the gonads via the hindgut (75). Subsequently, the differentiation of male gonads is initiated by the expression of Sex determining region of the Y-chromosome (*SRY*) gene, first resulting in specification of Sertoli cells and followed by Leydig cell differentiation (75). The somatic cells of the gonadal primordium start to differentiate into pre-Sertoli cells at approximately GW 7–8 (19, 20). The induction of SRY-related high-mobility group [HMG] Box 9 (SOX9) gene is the main driver of bipotential supporting cell differentiation into Sertoli cells. Subsequently, Sertoli cells proliferate, aggregate around the primitive germ cells and start tubulogenesis, i.e., the formation of testis cords (46). About 1 week after the Sertoli cells have been specified, Leydig cell differentiation is initiated in primitive interstitial cells of mesonephric origin. There is some evidence that this process might be controlled by the paracrine influences of AMH (19). During the second trimester of pregnancy, Sertoli cells proliferate rapidly, and the number of germ cells and Leydig cells also increases—adding to the volume of the testis (19).

The developing testis is anchored by two structures—the cranial suspensory ligament attaching the upper pole of the testis to the diaphragm and the genitoinguinal ligament (gubernaculum) anchoring the testis to the future inguinal area via the epididymis (77, 78). Testicular descent from the abdomen to the scrotum is a complex process that is divided into two phases—*transabdominal phase* and *inguinoscrotal phase* (23, 46).

*The transabdominal phase* occurs approximately during GW 10–15. Animal studies have shown that INSL3 from the testis has a role in inducing shortening of the proximal end of the gubernaculum (gubernacular cord) and enlargement of the distal end (bulb). These effects allow the gubernaculum to hold the testis at the inguinal area while the fetus is growing (79–82). Androgens are also probably needed in this phase since the rodents exposed to antiandrogen (flutamide) before or during the outgrowth phase of the gubernaculum display disrupted inguinoscrotal descent (83). AMH may also have a role in testicular descent by causing shortening of the gubernacular cord (84). The inguinal canal is formed around the gubernaculum from early fetal life, and enlargement of gubernaculum causes dilatation of the inguinal canal, facilitating later testicular descent (77, 85, 86). At the end of this phase, the testis is located near the deep inguinal ring. In rodents, cranial suspensory ligament (CSL) starts to regress due to androgen effect (80), but the role of CSL in testicular descent in humans is less clear (23, 75).

Most of the patients with cryptorchidism have disruption in the inguinoscrotal phase (46, 87, 88). The *inguinoscrotal phase* starts around GW 23–25 (75). Processus vaginalis, which is a diverticulum of the peritoneal membrane, covers the testis, epididymis, and gubernaculum. These structures move through the inguinal canal as one unit (85). Subsequently, the gubernaculum starts to shrink, leaving behind only a remnant called the scrotal ligament (86). Androgens have an essential role in this phase, and therefore subjects with androgen insensitivity syndrome often have disruption in this process (89, 90). Testosterone is important during the inguinoscrotal phase, which is in contrast to the early gestation when DHT, instead of testosterone, is critical for differentiation of male external genitalia (91, 92). In addition, an animal study has suggested that INSL3 and its receptor (RXFP2/LGR8) also have a role in the inguinoscrotal phase (93). By the end of this phase, i.e., around birth, the testis should be located in the scrotum (2, 46).

It is quite common that boys with cryptorchidism at birth undergo spontaneous testicular descent before the age of 6 months, or even before 3 months of age, i.e., during mini-puberty (14, 37, 94–96). A Finnish-Danish birth cohort study demonstrated a prevalence of cryptorchidism at birth of 2.4% in Finland and 9.0% in Denmark and the prevalence at 3 months was 1.0 and 1.9% and in Finland and Denmark, respectively (14). Spontaneous descent is, however, rare after 6 months of age (14, 94, 96). Interestingly, insulin-like growth factor 1 (IGF-1) has been implicated a role in postnatal testicular descent (33). We have previously shown that postnatal distance between testis and pubic bone positively correlates with testosterone/LH ratio, inhibin B/FSH ratio, and IGF-1 level at the age of 3 months (37). Testosterone/LH ratio and IGF-1 level significantly predicted the testis-pubic bone distance at the age of 3 months, but not at 18 months of age. In contrast, inhibin B/FSH ratio significantly predicted the distance at the age of 18 months, but not at 3 months. The results from this study suggest a role for Sertoli cell and Leydig cell function, and IGF-1 in post-natal testicular descent (37).

## Classification of Cryptorchidism

The majority of cryptorchid cases are detected at birth. This condition is called *congenital cryptorchidism*. However, some boys born with scrotal testes may later experience testicular ascent to a higher position, so-called *acquired cryptorchidism (ascensus testis, ascending testis*) (97). In contrast, *recurrent cryptorchidism* is a condition where the testis was undescended at birth but underwent spontaneous descent, subsequently followed by ascent back to a higher position (97).

### Acquired Cryptorchidism

A study in Denmark has shown that the prevalence of acquired cryptorchidism before 3 months of age is lower than after 3 months (0.2% at 3 months, 0.6% at 18 and 36 months) (97). This result is in agreement with a study in the UK, which showed that the prevalence of cryptorchidism was 5.9% at birth, 2.4% at 3 months, and 6.7% at 12 months (98). These findings support a role for mini-pubertal hormone surge in testicular descent. Even in normal boys, testes often descend after birth until 3 months and then ascend a little (37).

Acquired cryptorchidism complicates the interpretation of study results on congenital cryptorchidism in childhood or men in general for several reasons. First, in some studies no examination data are available at birth, especially if adult men are studied, therefore making distinction between this condition and congenital cryptorchidism more difficult. Moreover, acquired cryptorchidism has to be distinguished from *retractile testis*, which might be challenging. Second, patients may not be aware of this condition, and therefore the onset of acquired cryptorchidism is uncertain in most cases resulting in delay of treatment. Lastly, natural history and clinical consequences of acquired cryptorchidism are still unclear. However, evidence suggests that this condition has similar long-term adverse effects on testis as congenital cryptorchidism, as will be discussed below (99, 100).

## Etiologies of Cryptorchidism

Testicular descent is a complex process involving genetic, hormonal, anatomical, and environmental factors. Diseases and conditions affecting any of the associated factors can cause cryptorchidism. Several genetic disorders and syndromes are connected with cryptorchidism, so-called syndromic cryptorchidism. These conditions are characterized by decreased levels or actions of hormones involved in testicular descent, or associated with anatomical malformations (23, 101). Table 1 shows a list of conditions related to cryptorchidism. However, it is generally appreciated that cryptorchidism usually occurs as an isolated condition, and unassociated with any other congenital anomaly or syndrome, sometimes referred to as isolated or non-syndromic cryptorchidism (1, 120). Notably, the specific underlying causes in majority of cryptorchid cases cannot be identified (124). Below we discuss known conditions in which cryptorchidism is a common clinical manifestation, and different factors that are associated with cryptorchidism.

**Table 1:** Conditions associated with cryptorchidism.

**Conditions**	**Prevalence of cryptorchidism**	**References**
Diseases or syndromes associated with decreased androgen levels 1. Disorders of sex development (DSD)^*^ **1.1 Sex chromosome DSD** - 47,XXY (Klinefelter syndrome and variants) - 45,X/46,XY (mixed gonadal dysgenesis) - 46,XX/46,XY (chimerism) **1.2 46,XY DSD** **1.2.1 Disorders of testicular development** - *Complete or partial gonadal dysgenesis* Mutations in *ARX, ATRX, CBX2, DAX1* (*NROB1), DHH, DHX37, DMRT1, EMX2, ESR2, FGFR2, GATA4, HHAT, MAP3K1, NR5A1, SF1, SOX9, SRY, TSPYL1, WNT4, WT1*(WAGR syndrome, Denys-Drash syndrome, Frasier syndrome), *ZFPM2*, and *ZNRF3* genes **1.2.2 Disorders in androgen synthesis or action** - *Androgen biosynthetic defect* Abnormal LH (*LHB*) Steroidogenic acute regulatory protein (StAR deficiency) (*STAR*) 7-Dehydro-cholesterol desmolase deficiency (Smith-Lemli-Opitz syndrome) Cholesterol desmolase deficiency (*CYP11A1*) 3β-hydroxysteroid dehydrogenase type 2 deficiency (*HSD3B2*) 17,20-lyase deficiency or combined 17 hydroxylase/17,20-lyase deficiency (*CYP17A1*) P450-oxidoreductase deficiency (*POR*) 17β-Hydroxysteroid-dehydrogenase type 3 (*HSD17B3*) 5-alpha reductase type 2 enzyme deficiency (*SRD5A2*) Cytochrome b5 deficiency (*CYB5A*) Backdoor steroidogenetic enzyme deficiency (*AKR1C2, AKR1C4*)	27–37%	(102, 103) (104) (105) (106, 107)
- *Defect in androgen action* Partial androgen insensitivity (*AR*) Infantile onset X-linked spinal muscular atrophy - *LH receptor defects* Inactivating mutation of LH receptor gene (*LHCGR*) (Leydig cell hypoplasia, aplasia) 1.3 46,XX DSD^*^ - Ovotesticular DSD - Testicular DSD (e.g. *SRY*+, dup *SOX9, RSP01*) 46,XX male		
2. Congenital hypogonadotropic hypogonadism in 46,XY^**^: - *Isolated hypogonadotropic hypogonadism with anosmia (Kallmann syndrome)* *KAL1, FGFR1/FGF8, PROK2/PROKR2, KAL1, NELF, HS6ST1, WDR11*, and *SEMA3A* gene mutations *CHD7* gene mutation (CHARGE syndrome) - *Normosmic isolated hypogonadotropic hypogonadism* Mutations in *KISS1, GPR54, LEP, LEPR, TAC3/TACR3, and GNRH1/GNRHR* genes (Also reported mutations in *FGFR1/FGF8, PROKR2, CHD7*, and *WDR11* genes) - *Multiple pituitary hormone deficiencies* Mutations in *PROP1* genes, *HESX1* gene (Septooptic dysplasia) **3. Congenital hypergonadotropic hypogonadism** - Down syndrome - Noonan syndrome **4. Syndromes associated with both primary and secondary hypogonadism** - Prader-Willi syndrome - Bardet-Biedl syndrome	38–69.6% 50–70% 6.5% 65.7–80% 80–90% 11–13%	(108–110) (111) (112) (113–115) (116, 117) (118, 119)
**Conditions associated with decreased INSL3 or AMH levels or actions**		
*INSL3* or *RXFP2* mutations		
Persistent Müllerian duct syndrome: AMH and AMH receptor (*AMH, AMHR2*)		
**Other conditions related with cryptorchidism** - Aarskog-Scott - Acrocallosal - Acrodysostosis		(1, 120, 121)
- Adams-Oliver - Amyoplasia congenital disruptive sequence - Aniridia—Wilms tumor association - Apert - Autosomal recessive chondroplasia punctate - Beckwith-Wiedemann - Cardio-facio-cutaneous - Catel-Manzke - Chondroectodermal dysplasia - Cockayne - Coffin-Siris - Congenital microgastria-Limb reduction complex - DeLange - Deletion 3p, 4p, 5p, 9p, 11q, 13q, 18q, 22q11.2 - Diastrophic dysplasia - Distal arthrogryposis - Distichasis-lymphoedema - Dubowitz - Duplication 3q, 9p, 10q, 15q - Escobar - Ectrodactylia-ectodermal dysplasia clefting - Fanconi anemia - Femoral hypoplasia—unusual facies - Fetal hydantoin - FG - Fraser - Freeman-Sheldon - Frontometaphyseal dysplasia - Fryns - Gorlin - Greig cephalopolysyndactyly - Hallermann-Streiff - Jarcho-Levin - Johanson-Blizzard - Langer-Giedion - Larsen - Lenz-Majewski hyperostosis - Lenz Microphthalmia - Marden-Walker - Marshall - McKusick-Kaufmann - Meckel-Gruber - Meier-Gorlin - Miller - Miller-Diker - Myotonic dystrophy - Mowat-Wilson - Multiple lentigines - Neu-Laxova - Opitz G/BBB - Oto-palato-digital (type II) - Pallister-Hall syndrome - Pena-Shokeir phenotype - Peters'-plus - Pfeiffer - Poplietal pterygium - Prune-Belly syndrome - Posterior urethral valve - Roberts-SC phocomelia - Robinow - Rothmund-Thomsom - Rubinstein-Taybi		
- Saethre-Chotzen - Seckel - Senter-KID - Shprintzen-Goldberg - Simpson-Golabi-Behmel - Smith-Magenis - Toriello-Carey - Torticollis keloids cryptorchidism renal dysplasia - Treacher-Collins - Triploidy - Trisomy 8, 9 mosaic, 13, 18 - Ulnar-mammary - Weaver - X-linked α-thalassemia/mental retardation - XXY - XXXXY - XYY - Zellweger - 1p36 deletion		

The prevalence of cryptorchidism in each syndrome that has been reported in the literature is shown.

* Modified from the consensus statement on management of intersex disorders (121, 122).

** For a list of genes implicated in congenital hypogonadotropic hypogonadism, see (123).

*CHD7, Chromodomain helicase DNA binding protein; DAX1, Dosage-sensitive sex reversal congenital adrenal hypoplasia critical region on the X chromosome type 1; FGFR1, Fibroblast growth factor receptor type 1; GnRH1, gonadotropin releasing hormone; GnRH-R, GnRH receptor; GPR54, G-protein coupled receptor 54 (kisspeptide receptor); HS6ST1, Heparan sulfate 6-Osulfotransferase1; LEP, leptin; LEPR, leptin receptor; NELF, Nasal embryonic LHRH factor gene; NROB1, nuclear receptor subfamily 0, group B, member 1; PROK2, prokinecitin-2; PROKR2, Prokinectin receptor 2; SEMA3A 7, Semaphorin-3A; TAC3, gene encoding neurokinin B, TACR3, gene encoding neurokinin receptor; WDR11, WD repeat-containing protein (17)*.

### Sex Chromosome Disorders

Boys with abnormal sex chromosome number, e.g., 45,X/46,XY mosaicism, can have a wide range of external genitalia phenotypes from normal female, ambiguous (which can be asymmetric) to normal male phenotypes (17). The gonads may vary from streak gonads and dysgenetic testes to normal testes. The gonads are typically located along the normal route of testicular descent and while streak gonads tend to be at the intra-abdominal position, well-formed testes are preferentially located at the inguinoscrotal region (17).

Men with Klinefelter syndrome have supernumerary X chromosome, which is the cause of testicular failure (125). The majority of men with Klinefelter syndrome have poor Leydig cell function during mid-to-late puberty, and congenital cryptorchidism is rather common with a reported prevalence of 27–37% (102, 103). While the underlying mechanism is not known, androgen insufficiency during fetal development has been proposed. Notably, micropenis is a frequent finding in Klinefelter boys, and a small study has shown that amniotic total testosterone levels is in the female range in some boys with Klinefelter syndrome (125).

### Cryptorchidism in 46,XY Individuals

Since androgens have a crucial role in the inguinoscrotal phase of testicular descent, many diseases/syndromes related to male hypogonadism or decreased androgen action can manifest as cryptorchidism (2).

#### Disorders of Gonadal Development

Patients with partial gonadal dysgenesis have variable internal and external genitalia phenotypes. The gonads vary from streak gonads to dysgenetic testes, or they may regress (17, 126). Patients with ovotesticular DSD (disorder of sex development; 46,XX, 46,XX/46,XY, or 46,XY) have variable manifestations of gonads (testis, ovary, or ovotestis) and internal/external genitalia, and decreased gonadal function. The testis or ovotestis can be found at any location along the route of testicular descent (17).

#### Decreased Androgen Biosynthesis Due to Testicular Enzyme Deficiency

Deficiencies in any of the enzymes involved in testicular synthesis of testosterone or DHT result in decreased androgen production and action, which can manifest itself since fetal life. These enzyme deficiencies are typically inherited in an autosomal recessive manner (127). A list of enzymatic defects and gene mutations are shown in Table 1.

#### Hypogonadotropic Hypogonadism

Boys with congenital hypogonadotropic hypogonadism frequently present with cryptorchidism and/or micropenis. Gene mutations involved in isolated hypogonadotropic hypogonadism are listed in Table 1. Mutations in the coding regions of *FGFR1, GNRHR, PROK2, PROKR2, TAC3*, and *TACR3* genes can cause isolated hypogonadotropic hypogonadism, however, these mutations are not commonly found in patients with isolated cryptorchidism (128). A French study involving 46 boys with CHH reported that cryptorchidism was detected in 32 boys (69.6%)—eight boys with unilateral and 24 boys with bilateral cryptorchidism (110). A study of 98 Brazilian men with Kallmann syndrome reported that cryptorchidism was observed in 54% of all patients, 65% (11/17) of patients with *KAL-1* gene mutation (two boys with unilateral and nine boys with bilateral cryptorchidism) and 23% of patients with isolated hypogonadotropic hypogonadism (129). Importantly, placental hCG, not LH, controls androgen production during the first half of pregnancy when the transabdominal phase of testicular descent takes place (during GW10–15) and differentiation of male external genitalia occurs (18, 85, 130–132). This might explain why cryptorchidism is not found in all patients with hypogonadotropic hypogonadism (2), and these patients do not have ambiguous genitalia, as patients with testicular androgen synthesis defects. However, hypogonadotropic hypogonadism might be associated with testicular ascent during infancy (133). Patients with Prader-Willi syndrome have both central and primary hypogonadism, and almost all of them have unilateral or bilateral cryptorchidism at birth (134).

#### Decreased Androgen Action

In addition to male hypogonadism, patients with partial androgen insensitivity syndrome caused by androgen receptor gene mutation may also present with cryptorchidism (89, 90).

#### Genetic Defects in Genes Related to Hormones Controlling Testicular Descent

Patients with persistent Müllerian duct syndrome have normal male external genitalia, but have Müllerian structures (uterus, fallopian tubes, and upper vagina) (135). This is because Müllerian ducts do not regress during fetal life due to absence of AMH function. The syndrome is caused by an inactivating mutation in a gene encoding AMH or its receptor (*AMHRII* gene). Patients may present with bilateral cryptorchidism, unilateral cryptorchidism with contralateral hernia, or transverse testicular ectopia (135). The reason for cryptorchidism may be anatomical hindrance of testicular descent. However, there is also evidence that AMH may act on gubernaculum by shortening it (84). Interestingly, mice with *Amh* defects have normal testicular descent (136). Unlike AMH, INSL3 has a clear role in testicular descent in mice (79–81). However, *INSL3* and *LGR8* gene mutations are rare in cryptorchid patients (137–139).

#### Genetic Syndromes and Other Genetic Defects

Cryptorchidism may also occur as part of a genetic syndrome with unclear underlying mechanisms, including Down syndrome and Noonan syndrome. Boys with Down syndrome have an increased risk of both congenital and acquired cryptorchidism (112). Hypergonadotropic hypogonadism has been reported in some boys with Down syndrome and Noonan syndrome, which might explain the increased risk of cryptorchidism in the affected boys (112, 140). In addition, another proposed mechanism of acquired cryptorchidism in Down syndrome is an impaired migration of the spermatic cord along the path of the gubernaculum and impaired cord elongation (112).

#### Anatomical Defect of the Abdominal Wall, or Decreased Abdominal Pressure

Cryptorchidism is also found in other syndromes and defects not associated with androgen deficiency, including Prune belly syndrome, posterior urethral valve, and abdominal wall defects (121). This suggests that a mechanical defect is causing cryptorchidism (101).

#### Maternal Condition, Environmental Exposure, and Other Factors

Although several genetic disorders and syndromes have been associated with cryptorchidism, the specific causes in the majority of cryptorchid cases cannot be identified (124). Individual factors that have some association with cryptorchidism are low birth weight, small for gestational age, preterm birth, family history of cryptorchidism, maternal heavy cigarette smoking, caffeine consumption during pregnancy, endocrine-disrupting chemical exposure or medication use during pregnancy, maternal gestational diabetes, low parity, and paternal smoking during pregnancy (2, 121, 141, 142). These factors are postulated to disrupt fetal testicular development and cause testicular dysgenesis syndrome (TDS). According to the TDS hypothesis, some male reproductive disorders/manifestations, including cryptorchidism, hypospadias, lowered semen quality, testicular germ cell tumors, decreased serum testosterone level, and short anogenital distance, may share the same origin in fetal life (9, 10, 124, 143).

An endocrine-disrupting chemical (EDC) is an exogenous substance or mixture that modifies function(s) of the endocrine system and consequently causes adverse health effects in an intact organism, or its progeny, or (sub) populations (144). Some EDCs show anti-androgenic or estrogenic effect, or affect androgen or INSL3 levels or action during fetal development in animal studies (145). Animal studies have demonstrated that exposure to anti-androgen or estrogen during a critical period of male reproductive organ development during embryonic days 13.5–17.5 in rats (equivalent to GW 7–15 in humans) is related to the occurrence of cryptorchidism (9, 18, 146, 147). In humans, boys with a history of *in utero* exposure to diethylstilbestrol, a synthetic non-steroidal estrogen, have been shown to have a two-fold increased risk of cryptorchidism (148). Association between EDCs, such as dioxins, polybrominated flame retardants and pesticides, and cryptorchidism, has been reviewed in (149).

## Histological Changes in the Testis of Patients With Cryptorchidism

The histological changes in the cryptorchid testis include abnormalities in a variety of testicular cells. Number of germ cells per tubule were normal during the first year of life but subsequently decreases significantly, especially during 1–3 years of age (42, 150–154). Sertoli cell and germ cell numbers are lower in boys who have undergone orchiopexy at the age of 3 years than in those who were operated at the age of 9 months (42). During the first year of life, the number of Ad spermatogonia declined. The gonocytes disappeared from the cryptorchid testis by the age of 12 months instead of normal 6 months (152–154). This suggests an impaired transformation of gonocytes into Ad spermatogonia (152–154). A decreased number of Leydig cells was detected after the first few months after birth in cryptorchid boys (152, 153). In addition, interstitial or peritubular fibrosis appeared with a concomitant decrease in the number of germ cells, which worsened with increasing age in cryptorchid testes (155, 156). The descended testes of the boys with unilateral cryptorchidism also show histological abnormalities. These include a reduced number of Ad spermatogonia and a delayed development of primary spermatocytes (154).

## Reproductive Hormone Levels in Patients With Cryptorchidism

There are a number of studies exploring the HPT axis hormone levels in patients with cryptorchidism. Table 2 summarizes the subjects and the main findings of these studies.

**Table 2:** Summary of studies on reproductive hormone levels in boys with cryptorchidism and men with a history of cryptorchidism in childhood^*^.

**First authors**	**Study design** **Country of investigation** **Study subjects (*n*)**	**Age at hormone measurements, hormonal tests**	**Comparisons**	**Hormone results**
**At birth**				
Bay et al. (50)	Prospective study Finland and Denmark 3 groups of boys - transient cryptorchidism (cryptorchidism <3 months of age) - Persistent cryptorchidism (cryptorchidism persisted > 3 months of age) - Controls *Finland* - 21 transient cryptorchidism - 20 persistent cryptorchidism - 20 controls *Denmark* - 11 transient cryptorchidism - 1 persistent cryptorchidism - 26 controls	At birth	Cryptorchid vs. non-cryptorchid boys	Cord blood INSL3 level in Finnish boys with cryptorchidism was significantly lower than in controls. (non-significant difference in Danish boys)
Fénichel et al. (157)	Prospective case-control study France 26 boys with transient cryptorchidism 26 boys with persistent cryptorchidism 128 controls	At birth	Cryptorchid vs. non-cryptorchid boys	Cord blood INSL3 level in cryptorchid boys was significantly lower than in controls. LH, T, hCG, AMH, inhibin B, and SHBG levels: not different
**Mini-puberty**				
Gendrel et al. (158)	Longitudinal study France	Every month from the age of 1 to 4 months	Cryptorchidism with spontaneous testicular descent vs. persistent cryptorchidism	Plasma FSH was not different between 2 groups.
	57 term cryptorchid boys - 35 unilateral cryptorchidism - 22 bilateral cryptorchidism Cryptorchid boys classified into two groups: -Spontaneous testicular descent -Persistent cryptorchidism > 6 months			Plasma LH and T levels were significantly lower in persistently cryptorchid boys.
Baker et al. (159)	Case-control study United Kingdom Cases: 21 boys born preterm (mean gestational age of 30 weeks) - 11 boys with unilateral cryptorchidism - 10 boys with bilateral cryptorchidism Controls: 21 boys matched for gestational age, birthweight, duration of ventilation assistance and duration of phototherapy	Mean age of blood sampling -2 days 5 days weekly until hospital discharge (mean age of 67 days) Only plasma T levels were measured.	Cryptorchid vs. non-cryptorchid boys	At 2 days and after 6 weeks: Cryptorchid boys had significantly lower plasma T levels than controls.
De Muinck Keizer-Schrama et al. (160)	Longitudinal study The Netherlands Three groups of boys, aged <1 y: Cryptorchidism persisted > 1 y (*n =* 29) (untreated) Spontaneous descent within 1 y (*n =* 19) Controls (*n =* 160)	*At 3, 6, 12 months:* Basal and peak LHRH-stimulated serum LH and FSH Basal T *At 12 months:* Basal and post-hCG stimulation: T, DHT, and T precursors	Persistent vs. transient cryptorchidism vs. controls	Basal, peak LHRH-stimulated LH and FSH levels were not different between three groups, except basal serum LH in group 2 was higher than that of group 3. Basal and peak LHRH-stimulated serum LH and FSH levels had similar changes over time. Basal and post-hCG stimulated serum levels of DHT and T precursors were not different between three groups.
Hamza et al. (161)	Longitudinal study France and Egypt 84 boys with cryptorchidism (42 unilateral and 42 bilateral) No controls 1-year follow-up	Blood hormone levels at the age of 2–5 days 3 months 6 months	Unilateral vs. bilateral cryptorchidism	Blood FSH, LH, T levels of unilateral and bilateral cases were not different.
			Cryptorchidism with spontaneous testicular descent vs. permanent cryptorchidism	Boys with spontaneous testicular descent showed peak levels of LH and T at 2–3 months of age. FSH levels did not show the peak. Boys with permanent cryptorchidism: very low FSH, LH and T levels during study
Raivio et al. (162)	Cross-sectional study Finland Cryptorchid and non-cryptorchid boys (total *n =* 80)	At 3 months	Testis located at scrotal or high scrotal position vs. higher position or non-palpable	Testicular location of all boys with detectable serum androgen bioactivity was at scrotal or high scrotal position (*n =* 26). All boys with testis located at suprascrotal, or inguinal position or testes were non-palpable (*n =* 16) had undetectable serum androgen bioactivity. Boys with testis located in scrotal or high scrotal position (*n =* 23) had significantly higher serum T levels than those with higher testicular location or testes were non-palpable (*n =* 11).
Barthold et al. (163)	Case-control study USA 20 boys with non-syndromic cryptorchidism (15 unilateral cryptorchidism) 26 non-cryptorchid boys	Plasma levels: 2 months of age Urine samples: every month from the age of 7 days to 4 months	Cryptorchid vs. non-cryptorchid boys	No difference between the groups in the hormone levels in plasma levels of FSH, LH, total T, FAI, inhibin-B, estradiol, and SHBG or urinary levels of FSH, LH, T, and estradiol.
Suomi et al. (49)	Prospective cohort study Finland and Denmark Finland - 88 cryptorchid boys - 300 non-cryptorchid boys Denmark - 34 cryptorchid boys - 399 non-cryptorchid boys	At 3 months	Cryptorchid vs. non-cryptorchid boys	*Finnish boys*• Cryptorchid boys had significantly higher FSH, LH levels, FSH/inhibin B ratio and lower inhibin B levels. • T levels of the two groups were not different. *Danish boys*• Cryptorchid boys had significantly higher FSH level than non-cryptorchid boys. Other hormones were similar between the two groups.
Bay et al. (50)	Prospective study Finland and Denmark Finnish boys - 28 transient cryptorchidism - 51 persistent cryptorchidism - 100 controls Danish boys - 26 transient cryptorchidism - 11 persistent cryptorchidism - 51 controls	At 3 months	Cryptorchid vs. non-cryptorchid boys	Both countries: Serum INSL3, LH, T levels between cryptorchid cases and controls were not different. Serum LH/INSL3 was significantly higher in persistently cryptorchid boys than that of controls. Serum LH/T ratio was significantly higher in persistently cryptorchid boys than that of controls (only among Finnish boys). Serum levels of INSL3, LH, and T, and LH/INSL3 and LH/T ratios between transiently cryptorchid boys and persistently cryptorchid boys were not different.
Pierik et al. (164)	Case-control study The Netherlands 43 boys with cryptorchidism 113 controls	At 1–6 months	Cryptorchid vs. controls	Serum FSH, inhibin B, and AMH levels: not different between the 2 groups. Cryptorchid cases had significantly lower T and NSBT levels.
**Prepuberty**				
Gendrel et al. (165)	Cross-sectional study France 154 boys who had history of - Unilateral cryptorchidism (*n =* 64) or - Bilateral cryptorchidism (*n =* 90) 46 controls	1 month−15 y	LHRH test Cryptorchid boys vs. control levels	Peak LH levels in boys with history of cryptorchidism were significantly lower than controls (from infancy to early pubertal stage). FSH levels of boys with history of cryptorchidism and controls: no difference
			hCG stimulation test	Basal plasma T levels: no difference between cases and controls Peaked T levels: blunted in boys with a history of cryptorchidism from the age of 1 year until mid-puberty
De Muinck Keizer-Schrama et al. (160)	Described above			
Longui et al. (166)	Cross-sectional study Brazil Boys with a history of cryptorchidism, aged below 4 y (*n =* 11) Boys with a hypospadias (used as controls) (*n =* 8)	mean age: 2.2 y	Boys with a history of cryptorchidism vs. controls	Basal LH and T concentrations were not different between the 2 groups Boys with a history of cryptorchidism had significantly lower basal inhibin and higher FSH levels than controls. After hCG plus human menopausal gonadotropin (hMG) treatment, the inhibin-to-FSH ratio was significantly lower in boys with a history of cryptorchidism.
Christiansen et al. (167)	Cross-sectional study Denmark 62 boys with untreated cryptorchidism (45 unilateral and 17 bilateral cryptorchidism) 156 healthy, prepubertal boys	Median age 7.7 y, ranged from 4.1 to 13.6 y	Cryptorchid vs. non-cryptorchid boys	Basal inhibin B, T, FSH, and LH levels between cryptorchid cases and healthy controls: no difference
			Unilateral vs. bilateral cryptorchidism	Basal levels of inhibin B, T, FSH, and LH between boys with unilateral and bilateral cryptorchidism: no difference
			Hormone levels of cryptorchid boys after 3-week hCG injection (*n =* 18)	After hCG treatment in cryptorchid boys, T increased into the adult range and FSH and LH were suppressed.
Iwatsuki et al. (168)	Cross-sectional study Japan Four groups of boys: Surgical treatment for unilateral or bilateral cryptorchidism (*n =* 23) Hypospadias (*n =* 49) Cryptorchidism and hypospadias (*n =* 10) Hydrocele (*n =* 7)	At age: <12.5 y 12.5 to 13.5 y > 13.5 y and by Tanner stages	Compare four groups of boys	FSH levels in boys with both cryptorchidism and hypospadias was significantly higher than those of the other groups at ages 12.5–13.5 and >13.5 y, and during Tanner stages II and III. LH and T levels were not different among the groups.
Komarowska et al. (169)	Cross-sectional study Poland Boys with unilateral cryptorchidism (*n =* 105) Boys with inguinal hernia (controls) (*n =* 58)	Age 1–4 y	Boys with unilateral cryptorchidism vs. non-cryptorchid boys	Serum AMH, INSL3, and inhibin B of the two groups were not different.
Hamdi et al. (170)	Cross-sectional study France Cases: boys operated for cryptorchidism (*n =* 27) controls (*n =* 27) Age range: 14–32 months	Mean age Cases: boys operated for cryptorchidism: 26.6 months Control group: 24.4 months	Cryptorchid vs. non-cryptorchid boys	Serum inhibin B, AMH and testosterone levels of the boys operated for cryptorchidism were significantly lower than that of non-cryptorchid boys. (T levels were detectable in 10 cases and 10 controls.)
Grinspon et al. (171)	Retrospective, cross-sectional study Argentina Cryptorchid group Untreated bilateral cryptorchidism (*n =* 186) Untreated unilateral cryptorchidism (*n =* 124) Apparently normal boys (controls) (*n =* 179)	Median age 3 y (range 0.03–13.6 y)	Cryptorchid vs. non-cryptorchid boys	Median AMH standard deviation score (for age of normal boys) in the cryptorchid group was below 0.
			Unilateral vs. bilateral cryptorchidism vs. controls in each age group - 1–5.9 months - 6 months−1.9 y - 2–8.9 y - ≥9 y	Serum AMH level of boys with bilateral cryptorchidism was significantly lower than that of the unilateral cryptorchidism and control groups between the age of 6 months to 1.9 y and between 2 to 8.9 y. Serum FSH and LH levels were not different between the three groups at any ages.
**Puberty**				
Gendrel et al. (165)	Described in the pre-pubertal section			
Dickerman et al. (172)	Longitudinal study Israel 106 boys with cryptorchidism - unilateral (*n =* 77) - bilateral (*n =* 29) Follow-up at least two times per year from the age of 5–14 y (Mean age 11.6 y)	Plasma FSH and LH levels before and after LHRH test Plasma T level before and after hCG stimulation test The hormone levels were compared with normal range.	LHRH test: Unilateral vs. bilateral cryptorchidism vs. range for normal boys for chronological age at various pubertal stages according to data from previous studies	- Basal FSH level in boys with unilateral cryptorchidism during prepuberty and bilateral cryptorchidism at mid-puberty and full puberty: higher than normal reference range. - Basal LH in boys with bilateral cryptorchidism: higher than normal reference range. - Post LHRH test, FSH level in cryptorchid boys: higher than normal reference range. - Post LHRH test, LH level: higher than normal range in boys with unilateral cryptorchidism at prepuberty and at mid-puberty.
			hCG stimulation test: unilateral vs. bilateral cryptorchidism vs. range for normal boys for chronological age at various pubertal stages	- At the start of puberty: Unilateral cryptorchidid boys: basal and post-hCG stimulated T levels were higher than normal reference range. - At mid-puberty: basal T level in bilateral cryptorchidism group, aged 14–16 y, was lower than normal reference range. - At the end of puberty: basal T level in unilateral group was lower than normal reference range. T level after hCG stimulation was lower than normal reference range in cryptorchid boys.
**Adulthood**				
Lee and Coughlin (4)	Cohort study USA Men with a history of bilateral cryptorchidism (*n =* 8) Men with a history of unilateral cryptorchidism (*n =* 109) Control men (*n =* 53)	Adult age	Unilateral vs. bilateral cryptorchidism vs. controls	Men with a history of bilateral cryptorchidism had significantly lower inhibin B, significantly higher FSH and LH levels than men with a history of unilateral cryptorchidism and control men.
Brazao et al. (173)	Retrospective case-control study The Netherlands Group1: subfertile men with orchiopexy in childhood (*n =* 64, including 32 unilateral and 32 bilateral cryptorchidism) Group 2: non-cryptorchid subfertile men (*n =* 128) Group 3: fertile men (*n =* 32)	Median age: Group 1: 31 y Group 2: 33 y Group 3: 32 y	Subfertile men with orchiopexy in childhood vs. non-cryptorchid subfertile men vs. fertile men	Inhibin B levels of men in group 1 were significantly lower than those of men in group 2 and 3 FSH and LH levels in group 1 were significantly higher than those of group 2 and 3 FAI: no difference
Andersson et al. (174)	Cross-sectional Denmark 357 infertile men, including 72 men with a history of cryptorchidism (median age: 33 y) 318 fertile men, including 42 men with a history of cryptorchidism (median age: 30 y) Self-reported data	Adult age	Fertile vs. infertile men	Among both fertile and infertile men, history of cryptorchidism was associated with decreased serum inhibin B levels. Among infertile men, history of cryptorchidism was associated with higher LH level, lower T/LH ratio and lower estradiol level.
Rohayem et al. (175)	Retrospective case-control study Germany Men with a history of cryptorchidism with or without treatment (222 unilateral and 135 bilateral cryptorchidism) Men with no history of cryptorchidism and had normozoospermia and normal testicular size at adult age (*n =* 709) Age: 16–58 y	Mean age: Previous unilateral cryptorchidism: 34 y Previous bilateral cryptorchidism: 33 y Control men: 35 y	Men with vs. without a history of cryptorchidism	Mean FSH and LH levels: significantly higher in men with a history of cryptorchidism Mean total and free T levels: significantly lower in men with a history of cryptorchidism Estradiol: no difference
			Men with history of unilateral vs. bilateral cryptorchidism	Men with a history of bilateral cryptorchidism: significantly higher mean FSH and LH levels Mean total and free T, estradiol levels: no difference

* Some studies included subjects at different periods of life. The studies are described in the section, in which the main results are reported.

DHT, dihydrotestosterone; FAI, free androgen index; T, testosterone; y, year.

## At Birth

To our knowledge, two studies have investigated the cord blood levels of INSL3 in newborn boys with congenital cryptorchidism (50, 157). Bay et al. reported that levels of INSL3 in cord blood of Finnish, but not Danish, cryptorchid boys were significantly lower than in control boys. However, the Danish data may suffer some limitations due to a small number of subjects (50). Low INSL3 levels in cord blood of cryptorchid boys was also reported in a French study (157). LH, testosterone, hCG, AMH, inhibin B, and sex hormone-binding globulin (SHBG) concentrations of the cryptorchid and non-cryptorchid boys were similar (157). The low INSL3 level at birth could be a cause or a consequence of cryptorchidism—it might suggest that the cause of cryptorchidism is low secretion of INSL3 or indicate that mild Leydig cell dysfunction can be identified already at the early life of the cryptorchid boys. Sertoli cell function in cryptorchid testes seems to be normal at birth.

## Mini-Puberty

A number of studies have investigated HPT axis hormone levels in cryptorchid boys. However, there are some differences in the findings among the studies as described below.

### Sertoli Cell Function

Impaired Sertoli cell function in cryptorchid boys as compared with non-cryptorchid boys has been observed since the mini-pubertal period in one study (49), but not in the others (163, 164). A birth cohort study conducted in Finland and Denmark found higher FSH and lower inhibin B levels during mini-puberty in Finnish cryptorchid boys than in healthy controls. In Denmark, cryptorchid boys had higher FSH than non-cryptorchid boys but inhibin B levels were similar (49). These results suggested an impaired Sertoli cell function among cryptorchid boys (49). A strength of this study is that it is a follow-up of a prospective birth cohort with a harmonized research protocol. However, the blood was collected at the age of 3 months in both countries, which might not attain the peak of the mini-pubertal hormone surge, as it may occur already somewhat earlier (176). In contrast to the results of the Finnish and Danish birth cohort study, a case-control study involving 1-to-6-month-old boys in the Netherlands did not show significant differences in serum FSH, inhibin B, or AMH levels between the cryptorchid and control groups (164). Similarly, a case-control study in the USA showed that the levels of serum and urinary testosterone, estradiol, LH and FSH, and plasma levels of inhibin B and SHBG of the cryptorchid and non-cryptorchid boys were not different (163).

### Leydig Cell Function

Mild Leydig cell dysfunction in cryptorchid boys during mini-puberty has been demonstrated. Low testosterone levels or decreased post-natal testosterone peak has been reported in a number of studies, especially when patients with severe, persistent cryptorchidism were compared with milder cases (49, 158, 159, 161, 164, 177). A study in Finland and Denmark found higher LH level, LH/testosterone, and LH/free testosterone, but similar testosterone levels in cryptorchid boys as compared to controls, suggesting mild Leydig cell dysfunction with compensatory response by the pituitary gland (49). Boys with persistently severe cryptorchidism (one or both testes found at the suprascrotal or inguinal area or non-palpable at the age of 3 months) had significantly lower testosterone levels than boys with mild cryptorchidism (high scrotal position) with non-significantly higher LH levels (49). The LH/INSL3 ratio and LH/testosterone ratio were higher in persistently cryptorchid boys than in controls, however, INSL3 levels of the two groups were similar (50). When compared to boys with at least one testis located in a suprascrotal or a higher position at 3 months of age, boys with scrotal or high scrotal testes had higher androgen bioactivity (162), which is a measure of androgen action at the cellular level by using sensitive *in vitro* receptor bioassays (178). Leydig cell failure becomes evident in persistently severe cryptorchidism and the study subjects are likely analyzed with primary defects in Leydig cells, rather than Leydig cell dysfunction secondary to prolonged malposition of the testis (49, 50, 162).

In contrast, some studies have shown low testosterone and low LH levels in cryptorchid boys suggesting pituitary gland dysfunction (158, 161, 164). A study conducted in Egypt and France reported that the peak plasma LH and testosterone levels were low in persistently cryptorchid boys as compared to boys with spontaneously descended testis (161). A Dutch study demonstrated that cryptorchid boys had a lower testosterone and non-SHBG-bound testosterone (NSBT) than those of controls, whereas serum levels of SHBG, LH, FSH, AMH, and inhibin B were not different between the two groups (164). However, the authors reported that the proportion of boys with testosterone levels under the limit of detection was higher among cryptorchid boys aged 100 days or above than among controls. This finding might explain the lower testosterone and NSBT levels among cryptorchid boys as compared to the controls in this study. A study of preterm boys in the UK also found reduced peak testosterone levels in 1–2-month-old cryptorchid boys as compared to healthy controls (159). While there is clear evidence supporting decreased androgen levels in the mini-pubertal cryptorchid testis, yet two studies did not find any differences in the HPT axis hormone levels during mini-puberty between cryptorchid and non-cryptorchid boys (160, 163).

In conclusion, during mini-puberty Leydig cell function seems to be preserved in mildly and transiently cryptorchid boys but impaired in more severe and persistent cases. On the other hand, studies on Sertoli cell function have given inconsistent results. Only one study found a decreased Sertoli cell function as shown by lower inhibin-B and higher FSH levels.

The differences in the results of the reproductive hormone levels during mini-puberty may vary depending on the age at diagnosis, age at blood sampling (whether they were at the peak of HPT axis hormone secretion or not), severity of cryptorchidism, possible inclusion of retractile testis, or spontaneously resolved cases, syndromes associated with hypogonadotropic hypogonadism, size of the studies and sensitivity of the hormone assays. There are only a few studies that have reported the position of the testis, thus also indicating the severity of cryptorchidism or the underlying conditions associated with cryptorchidism, such as hypogonadism (49, 50, 162, 163).

## Prepubertal Period

The HPT axis becomes inactive ~6 months after birth in boys before its reactivation at the onset of puberty. During this prepubertal period, the levels of FSH, LH, and testosterone measured by routine laboratory methods are very low or undetectable in normal boys (56). Therefore, the measurement of basal levels of these hormones during this period is generally not useful for evaluation of the HPT axis function. Endocrine stimulation tests, including hCG stimulation test and GnRH test, are required for the assessment of presence of a testis and the function of prepubertal Leydig cells therein (179). In contrast, serum inhibin B and AMH levels are still detectable during this period in normal boys, which reflects the function of Sertoli cells (57, 180).

Pituitary gonadotroph function can be assessed by GnRH [also called luteinizing hormone-releasing hormone (LHRH)] stimulation test, and Leydig cell function can be evaluated by hCG stimulation test (179, 181). hCG is a glycoprotein hormone primarily produced by the placenta (179). It has a similar structure to LH and shares the same receptor as LH (LHCG receptor; LHCGR) on the Leydig cells. Compared to LH, hCG has 24 additional amino acids at the carboxy-terminal end, which increases its biological effect, plus a sialic acid terminal on a carbohydrate chain makes its half-life longer. As a result, hCG can sustainably stimulate steroid hormone production and can be used to test Leydig cell steroidogenic capacity (179). GnRH upregulates GnRH receptors on gonadotrophs and the expression level of gonadotropin subunit genes in the pituitary (181). A single intravenous injection of GnRH at a dose of 2.5 μg/kg stimulates LH, and to a lesser extent, FSH secretion from the anterior pituitary gland. Subcutaneous GnRH administration is less frequently used. The peak LH response to GnRH administration is used for the assessment of pubertal status (182). The peak LH and FSH levels are higher in peripubertal boys than those of the prepubertal boys (183). In prepubertal boys, the LH and FSH levels increase two- to four-fold after GnRH administration as compared to the basal level, and the peak LH/FSH ratio is about 0.7. Post-pubertally, LH level increases 6- to 10-fold, and FSH increases four- to six-fold after GnRH administration, and the mean peak LH/FSH ratio is about 3.5 (184). A blunted FSH and LH response suggests hypogonadotropic hypogonadism—either pituitary or hypothalamic dysfunction (181, 183). A sensitive gonadotropin assay, such as an immunofluorometric assay, is needed for reliable and sensitive testing (185). During prepuberty the GnRH test is not very informative because gonadotropin levels do not yet increase at this age, and therefore, differential diagnosis between CHH and a constitutional delay of puberty is difficult (186).

In summary, studies on Leydig cell function during the prepubertal period rely on the hCG-stimulated testosterone levels, while Sertoli cell function can be assessed by measuring basal inhibin B and AMH levels.

### Sertoli Cell Function

The basal levels of FSH and inhibin B of cryptorchid and non-cryptorchid boys have been found similar in a handful of studies (160, 165, 167). One study also reported no difference of these basal hormone levels between boys with unilateral and bilateral cryptorchidism (167). FSH levels after the GnRH test were similar between boys with transient cryptorchidism (spontaneously descended testis within 1 year of life), persistent cryptorchidism, and the controls, suggesting normal secretion of FSH in cryptorchid boys (160).

However, other studies have found lower inhibin B and AMH levels at the age of 2 years (170), lower AMH level between the age of 6 months to 8.9 years (171), and lower inhibin B and higher FSH levels at the mean age of 2 years in the cryptorchid boys as compared to controls, suggesting impaired Sertoli cell function (166). A study conducted in Japan demonstrated that FSH levels were higher in boys who had both cryptorchidism and hypospadias as compared to boys with cryptorchidism only, boys with hypospadias only and controls at the age of more than 12.5 years (168). The boys who had only cryptorchidism or only hypospadias had comparable FSH level as the controls. This might suggest that combined cryptorchidism and hypospadias are associated with a more severe testicular dysfunction, which supports the TDS hypothesis (168).

### Leydig Cell Function

Basal LH and testosterone levels, if still detectable, were found to be similar in cryptorchid and non-cryptorchid boys in some studies (160, 166–168). Other studies found that basal testosterone and LH levels were lower in cryptorchid boys, suggesting pituitary dysfunction in cryptorchid boys (170, 177). However, LH levels after the GnRH test between boys with transient cryptorchidism, persistent cryptorchidism, and controls were similar, which did not support the concept of pituitary failure in cryptorchid boys (160). It is worth noting that the gonadotropin responses in the GnRH test start to increase around the time of onset of puberty which might bias the results. Three studies compared testosterone levels following the hCG stimulation test between cryptorchid cases and controls. One study reported that the levels of the two groups were not different (160), but other two studies found blunted testosterone response after hCG stimulation (165, 177).

In conclusion, the available literature on Sertoli cell function in cryptorchid testis during prepuberty is inconsistent. While most studies suggest that Sertoli cell function is normal during prepuberty, some studies argue against it. Leydig cell function is generally regarded normal during this period.

## Puberty

The puberty in males starts when the testicular volume exceeds 3 mL, as assessed by an orchidometer (187–189). This co-occurs with increased levels of serum gonadotropins, testosterone, and inhibin B (58, 72, 190–195). Only two old studies have explored the reproductive hormone levels in cryptorchid boys during puberty (165, 172).

A longitudinal study published in 1979 followed a group of boys with a history of unilateral (*n* = 77) and bilateral (*n* = 29) cryptorchidism from the mean age of 11.6 years (range 5–14 years) to the time of full pubertal maturation (172). The hormonal data of these boys were compared to the normal range of hormones for chronological age at each pubertal stage. The study found that the basal and GnRH-stimulated FSH and LH levels in boys with a history of cryptorchidism were higher than normal during puberty, and the basal and hCG-stimulated testosterone levels were lower than normal after mid-puberty (172).

A 1977 study reported the results of a GnRH test and hCG stimulation test in 154 boys with cryptorchidism (64 unilateral and 90 bilateral), aged 1 month to 15 years (165). Plasma LH levels before and after the GnRH test were normal (165). However, the rise of plasma testosterone after hCG stimulation was reduced until mid-puberty. There was a positive correlation between testosterone level after the hCG stimulation test and LH level before and after the GnRH test (165).

## During Adulthood

It has been clearly shown that men with a history of cryptorchidism have an increased risk of infertility and TGCTs (6, 12), however, studies showing a long-term impact on hormonal levels in adulthood are more limited, and the results are not consistent.

### Sertoli Cell Function

Studies have reported higher levels of serum FSH or lower inhibin B or both in men with a history of cryptorchidism when compared to non-cryptorchid men, which suggests impaired Sertoli cell function (4, 173–175). Men with a history of bilateral cryptorchidism have lower sperm concentration and inhibin B levels, but higher FSH and LH levels than those in the unilaterally cryptorchid or control group (4).

### Leydig Cell Function

Some studies have found significantly higher serum LH and lower testosterone/LH ratio in men with a history of cryptorchidism as compared to controls, whereas testosterone levels were normal, indicating mild Leydig cell dysfunction with a compensatory pituitary response (173, 174). Yet, other studies have reported low testosterone and high LH concentrations in men with a history of cryptorchidism, which suggested a more severe Leydig cell dysfunction (4, 73, 175). Impaired Leydig cell function seems to be more related with bilateral cryptorchidism as shown in one study which found lower testosterone levels in men with a history of bilateral cryptorchidism than in men with a history of unilateral cryptorchidism (173).

A possibility that some men with acquired cryptorchidism have been included in the studies on adult reproductive health cannot be ruled out, and particularly data from retrospective studies might be prone to recall bias. Men with a history of unilateral acquired cryptorchidism were found to have significantly smaller testes (both the previously undescended testis and the contralateral descended testis), lower sperm concentration and lower percentage of sperm motility than controls (196). FSH, LH, testosterone, and inhibin B levels were similar between men with previously unilateral acquired cryptorchidism and controls. Interestingly, sperm concentration, percentage of progressive sperm motility, serum FSH, LH, testosterone, and inhibin B levels between men with a history of unilateral acquired cryptorchidism or unilateral congenital cryptorchidism do not differ (196). These parameters were also not different between men with bilateral acquired and bilateral congenital cryptorchidism (196).

In summary, current evidence suggests that cryptorchid boys have rather normal Sertoli cell and Leydig cell function at birth. The function of Sertoli cells starts to decline from the mini-pubertal period until adulthood as shown by decreased serum inhibin B and elevated FSH levels. Leydig cell function is preserved in boys with mild and transient cryptorchidism, but the function can be impaired in boys with severe and persistent cryptorchidism. Leydig cell function starts to decline from mid-puberty onwards. Some studies showed compensated Leydig cell function in adulthood as shown by normal serum testosterone but elevated serum LH level, which is worse in bilateral cryptorchidism than in unilateral cryptorchidism. However, there is likely variation in Leydig cell function given the fact that some studies demonstrated more severe functional failure of Leydig cells.

## Pubertal Development of Boys With Cryptorchidism and Testicular Size in Adulthood

Age at pubertal onset of boys with a history of cryptorchidism is not different from that of the healthy boys (189, 197), which is also supported by another study showing that testosterone level was not low at the start of puberty in cryptorchid boys (172). A Danish study found that self-reported signs of pubertal development of cryptorchid boys from the age of 11.5 years to the end of puberty or until the age of 18 years were not different from controls (198). However, a Finnish study found that age at the first self-reported conscious semen ejaculation was higher in boys with a history of cryptorchidism than in controls (197).

Seminiferous tubule compartment, composed of Sertoli cells and germ cells, accounts for 80–90% of testicular volume of adult human testis (199). Thus, testicular size of adult men reflects the reproductive capacity. In boys with a history of unilateral or bilateral cryptorchidism, the prepubertal growth of previously undescended testes does not differ from controls, however, at the end of puberty these testes are found smaller (189). The descended testis of boys with a history of unilateral cryptorchidism or monorchid testis, on the other hand, were bigger than that of the controls before puberty, but are found similar in volume at the end of puberty (189). In individuals with unilateral cryptorchidism, the previously undescended testis was smaller than the contralateral, normal testis from puberty to adulthood (172, 200). Interestingly, the testicular volumes of the operated testes and spontaneously descended testes were not different among boys with a history of unilateral cryptorchidism (189), which suggests that the testes were damaged in both conditions. However, a randomized treatment study showed that early orchiopexy is beneficial for prepubertal testicular growth (201). When testicular growth is monitored in boys with a history of congenital unilateral cryptorchidism, testicular volume of the boys who underwent orchiopexy at the age of 9 months increased significantly from the ages of 6 months to 4 years, whereas no testicular growth was observed in boys who underwent orchiopexy at 3 years (201). The spontaneously descended testes of the boys with congenital unilateral cryptorchidism were smaller than the contralateral, scrotal testes at all ages during a follow-up from birth to 5 years of age (202).

Studies in the adult men showed that testicular volume of subfertile cryptorchid men was smaller than that of non-cryptorchid subfertile men or fertile men from the general population (173). Somewhat surprisingly, the mean testicular volume of men with a history of bilateral cryptorchidism was bigger than that of formerly unilateral cryptorchid testes (200). However, both were smaller than the mean volume of normally descended testes of men with a history of unilateral cryptorchidism (200). A study in men with a couple infertility found that bi-testicular volume of the men with a history of unilateral cryptorchidism was bigger than that of the men with a history of bilateral cryptorchidism (175).

## Potential Mechanisms of Cryptorchidism-Induced Hypogonadism

To date, the mechanisms of hypogonadism in cryptorchidism are still elusive. There is evidence suggesting that from mini-puberty until adulthood serum inhibin B is lower and serum FSH level is higher in cryptorchid boys than in normal boys. During prepuberty, AMH levels are also lower in cryptorchid boys. This suggests an abnormal function in the testis rather than a defect in the hypothalamus or pituitary gland. Studies on Leydig cell hormone levels show contradictory results. Leydig cell hormone secretion seems to be better preserved in cryptorchidism than Sertoli cell function, suggesting that Leydig cells might be more tolerant of the abnormal testicular position than Sertoli cells. However, some studies in mini-puberty and adulthood have also shown elevated LH levels accompanied with normal testosterone levels, suggesting compensated Leydig cell dysfunction. Furthermore, a few studies have shown high LH and low testosterone levels, indicating a severe Leydig cell failure.

One proposed mechanism behind the adverse effects of cryptorchidism is the higher ambient temperature in the undescended testis. Temperature in the scrotum is 33°C, which is about 4°C lower than the core body temperature. Ambient scrotal temperature of around 33°C is seminal for the function of the testis after birth (203, 204). Higher temperature is considered to impinge especially on germ cells and it has been suggested that gonocytes require a lower ambient temperature for maturation (205). However, the importance of temperature on Sertoli cell and Leydig cell function is still unclear.

In men with unilateral cryptorchidism, the contralateral descended testis is also characterized by abnormal development (206) and has an increased risk of testicular cancer (207). This can be related to the underlying cause of cryptorchidism and the shared risk factors of testicular cancer in both testes. These findings also support the TDS hypothesis that was described earlier.

## Factors Affecting Reproductive Hormone Levels and Testicular Size in Patients With Cryptorchidism

### Age at Treatment

There is some evidence showing that early orchiopexy in cryptorchid patients is associated with higher serum inhibin B and/or lower FSH levels, suggesting better Sertoli cell function, as compared with late orchiopexy (before vs. after 2 years of age in a US study, before vs. after 5 years in a Dutch study and before vs. after 8 years in a Slovenian study) (73, 173, 208). Early orchiopexy was also associated with higher sperm concentration (173). A large case-control study conducted in Germany reported that age at correction of cryptorchidism (including patients treated with orchiopexy, hCG, or GnRH analogs) positively correlated with serum FSH and LH and negatively with testicular volume and sperm concentration (175). There was no correlation between age at correction of cryptorchidism and serum testosterone level (175). This is consistent with another study that found no correlation between age at orchiopexy and salivary testosterone level in men with a history of bilateral cryptorchidism (209). However, a study of unilateral cases in the US found an inverse correlation between age at orchiopexy and serum total testosterone level (210). Another study did not show the relationship between age at orchiopexy and serum hormone levels at the adult age (211). However, the results were not presented in detail, and e.g., correlation coefficients were not reported (211).

Boys who underwent orchiopexy and testicular biopsy at the age of 9 months had higher numbers of germ cells and Sertoli cells and larger testicular volume than boys who were operated and biopsied at 3 years of age (42). The spontaneously descended testes had similar testicular size at follow-up. Testicular volume at the time of operation correlated positively with the number of germ cells and Sertoli cells in the group treated at 9 months of age and it also associated positively with the number of Sertoli cells, but not germ cells, in the group treated at 3 years of age (42). The number of germ cells at 3 years of age was very low, therefore they do not contribute much to the testicular volume (42). In another study, increased duration of undescended testis was significantly associated with increased depletion of germ cells (OR 1.02, for each month) and Leydig cells (OR 1.01, for each month) (212). A systematic review and meta-analysis in 2018 showed that boys who underwent orchiopexy before the age of 1 year had 0.06 mL larger testicular volume (95% CI 0.01–0.10) and higher number of spermatogonia per tubule [mean difference 0.47 (95% CI 0.31–0.64)] than those operated after 1 year of age (213). After orchiopexy in boys with unilateral cryptorchidism, serum inhibin B increased, and serum FSH decreased, suggesting an improved Sertoli cell function (214).

Taken together, early orchiopexy is associated with a better outcome for Sertoli cell function and higher number of germ cells. However, the benefits of this practice for Leydig cell function, as shown by testosterone and LH levels, are controversial.

### Unilateral or Bilateral Diseases

The testicular hormone levels in bilateral cryptorchidism seem to be lower than those in unilateral cryptorchidism. Studies have revealed that men who were operated for bilateral cryptorchidism had higher FSH and LH levels and lower inhibin B levels than men who were operated for unilateral cryptorchidism or the controls, while testosterone levels were comparable between the groups (4, 211). A study conducted in Argentina found that risk factors for low AMH levels (less than third percentile) during prepuberty were bilateral cryptorchidism and the coexisting micropenis (171).

### Histology of Testis at Orchiopexy

A US study investigated serum reproductive hormones and semen analysis in adult men with a history of cryptorchidism who underwent orchiopexy and testicular biopsy of both testes (91 unilateral and 19 bilateral cryptorchidism) (215). Men who had a history of unilateral cryptorchidism and 0.1 or less Ad spermatogonia per tubule had significantly higher FSH levels than unilateral cryptorchid men with more than 0.1 Ad spermatogonia per tubule. However, this finding was not significant in bilaterally cryptorchid men, possibly due to a small number of subjects with a history of bilateral cryptorchidism (215).

### Testicular Location Before Orchiopexy

Testicular location of unilateral cryptorchidism before orchiopexy had no influence on paternity rate, duration of attempted conception before achieving paternity, sperm count, testosterone, or FSH levels in a study of 103 men with a history of unilateral cryptorchidism (216). A history of inguinal cryptorchidism was associated with significantly lower adult inhibin B level than that of other locations, and a history of ectopic testis was associated with higher LH level than that of other locations (216). Men with a history of intra-abdominal testes had a slightly increased risk for infertility (216). Again, a limitation of this study is the small number of cryptorchid men representing each testicular location, which possibly affects the statistical power of the study. In general, the results from this study did not support the idea that testis at a higher location is associated with poorer hormonal function.

## Effects of Cryptorchidism on Fertility

It has been clearly demonstrated that cryptorchidism has an adverse effect on fertility in adulthood (12). Paternity rate, as defined as the proportion of men who had fathered children or had attempted for more than 12 months, was significantly lower in men with formerly bilateral cryptorchidism than that of men with formerly unilateral cryptorchidism, or controls (12). The rates of successful paternity were 65.3 vs. 89.7 vs. 93.2%, respectively (12). Cryptorchidism is associated with an increased germ cell loss and resultant impaired fertility which become the worse the longer the testis stays in the undescended position (13). In normal boys, the maximum number of germ cells is found at ~3 months of age, then the number decreases significantly during the first 3 years of life in both cryptorchid testis, and to a lesser degree, normally descended testis (35, 42, 217). Therefore, the current guidelines for the management of cryptorchidism recommend orchiopexy before the age of 12 or 18 months (16, 218, 219).

Sperm concentration and sperm motility of men with childhood treatment of bilateral cryptorchidism were lower than those of men who were treated for unilateral cryptorchidism (208). Among men with a history of bilateral orchiopexy in childhood, serum FSH level was negatively associated with sperm concentration and serum inhibin B level (208). Serum inhibin B level had a negative correlation with FSH level (208). A study conducted in Finland found that the proportion of men who had normal sperm concentration was higher in men treated in childhood (by orchiopexy ± hCG) for unilateral cryptorchidism than that of men treated for bilateral cryptorchidism (90 vs. 50%, respectively) (220). All of the men who were treated for bilateral cryptorchidism before the age of 4 years had normal sperm concentration according to the WHO criteria (220), showing some benefits of early treatment.

## Role of Hormonal Treatment in Cryptorchidism

Induction of testicular descent in cryptorchid boys by intramuscular hCG injection or intranasal GnRH (LHRH) administration has been used. This practice is based on the concept that androgens are crucial for testicular descent in fetal life, and testosterone surge during mini-puberty has a role in the transformation of gonocytes into Ad spermatogonia, which are stem cells for future spermatogenesis (221). Notably, testosterone levels increased significantly after the administration of hCG or GnRH (222).

Although there might be some room for hormonal treatment in the management of cryptorchidism, studies on treatment of cryptorchidism with hCG or GnRH have demonstrated low success rates and a high rate of testicular re-ascent (223–225). Meta-analyses of randomized, blinded studies on hCG treatments showed a success rate of 19% (224), whereas GnRH studies showed a 21% success rate. These results represent a marginal improvement when compared to the placebo (4–6% success rate) (16, 224, 226). Notably, the reported success rates of orchiopexy have been more than 70% in most studies (218, 225). A more recent meta-analysis of hCG for cryptorchidism treatment in 2018 included seven randomized controlled trials, which assessed the efficacy of treatment with parenteral hCG as compared with intranasal GnRH or placebo (227). Different doses and regimens of hCG and GnRH were used. hCG induced complete testicular descent in 0–50.8% of patients with unilateral cryptorchidism and 0–22.4% of patients with bilateral cryptorchidism (227). The Nordic consensus published in 2007, European Urology guideline in 2016, and the American Urology Association guideline in 2014 do not recommend hormonal treatment for inducing testicular descent in cryptorchid patients and recommend orchiopexy as the preferred method of treatment (16, 218, 219). This is due to low efficacy of hormonal treatments, lack of long-term data, poor quality of studies, varied treatment protocols, and different study populations (16, 218, 219). Also, there are reports on an increased germ cell apoptosis after discontinuation of hCG treatment and possible harmful effects on germ cells (228–230). The adverse effects of hormonal treatments are common (in about 75% of the boys), although mostly mild and can decrease after discontinuation of the treatment (218). These include increases in scrotal redness and pigmentation, scrotal rugae, amount of pubic hair, penile and testicular size, and behavioral changes, including aggressiveness (222, 225).

In some studies, hormonal treatments, particularly GnRH, have been used before, but also after orchiopexy or orchiolysis (231). The aim has been to improve fertility potential in addition to the orchiopexy (231). This is based on the assumption that the hormonal surge during mini-puberty has a role in the transformation of gonocytes into Ad spermatogonia, which has been suggested to be important for male fertility (221, 232). The number of A dark spermatogonia from testicular biopsy at the time of orchiopexy was reported to predict the risk of infertility in adulthood (221, 232). Some studies of GnRH administration, before (in most studies) or after orchiopexy or orchiolysis, showed increased fertility indices, as assessed by the number of germ cells or spermatogonia per tubular cross-section or percentage of normal histology from testicular biopsy in childhood, or semen quality (16, 231, 233, 234). To date, there is only one study that reported a long-term effect of hormonal treatment on semen quality in adulthood (235), therefore the combined or sequential surgical and hormonal treatment cannot be recommended as routine practice at this time.

## Hormonal Treatment in Patients With Congenital Hypogonadotropic Hypogonadism During Infancy

Congenital hypogonadotropic hypogonadism (CHH) is a condition characterized by a decreased production, secretion, or action of GnRH (123). The patients with this condition lack FSH and LH and display the resultant low testosterone level since fetal life, and therefore they can present with micropenis or cryptorchidism at birth (108, 236, 237). Low testosterone levels during puberty and adulthood result in absent or incomplete puberty, eunuchoid body proportions, decreased virilization, reduced libido, and sexual function, infertility, or osteoporosis from long-standing hypogonadism (123).

Additionally, CHH patients lack gonadotropin and testicular hormone surge during mini-puberty giving the window of opportunity of early diagnosis of this condition in infancy, which can be performed by detecting low serum FSH, LH, testosterone, and inhibin B as compared with the reference ranges at 4–8 weeks after birth (123). Early diagnosis allows pediatricians to plan for induction of puberty at the appropriate time preventing the potential pubertal delay (108). Since the mini-pubertal hormonal surge is associated with Sertoli cell proliferation (238), the lack of mini-puberty might relate to reduced Sertoli cell proliferation and the resultant decreased fertility later in life (237). Therefore, some studies investigated the role of replacement of deficient mini-pubertal hormones by using GnRH, or gonadotropins during infancy in patients with CHH to improve reproductive health, especially fertility, later in life (237). However, only some of these studies included cryptorchid boys, and testicular descent was not the primary outcome of most of these studies, making it difficult to assess the effect of this approach on testicular descent (41, 133, 239–243). As far as we know, only two small studies in France and Greece have investigated the effects of gonadotropin administration during infancy in boys with CHH associated with cryptorchidism, and aimed at induction of testicular descent, or had testicular descent as one of the primary outcome (244, 245). These two studies used subcutaneous injection or infusion of rhFSH and rhLH in CHH infants with congenital cryptorchidism for 3 months in one study and 6 months in the other (244, 245). The treatment increased LH, FSH, inhibin B, and T levels to the normal or supranormal range. Complete testicular descent occurred in most patients. Their testicular size was normal. However, a few boys experienced testicular re-ascent and needed orchiopexy (244, 245). In Greece, the boys were followed 3–10 years after treatment. At the last visit, all testes were still in the scrotal position, but the size measured by the Prader orchidometer was marginally reduced, from a mean of 1.5 (1.0–2.5) mL during treatment to 1.0 (0.5–2.0) mL at the last visit (244). In a small study in Finland, five CHH boys with cryptorchidism were treated with rhFSH and testosterone enanthate during infancy (242). All of them underwent orchiopexy at the age of 2.0 ± 0.7 years. Serum inhibin B levels increased after starting the hormonal treatment and decreased after discontinuation of the treatment for 1.2 ± 0.4 months. During adolescence, the inhibin B levels were available in three boys (10.0–12.8 years of age), which were compared with six untreated CHH boys with a history of cryptorchidism (12.7–17.8 years of age). The inhibin B levels between these two groups were not different. The results of this study suggested a transient effect of the combined rhFSH and testosterone treatment on Sertoli cell function (242). Table 3 summarizes the studies on hormonal treatment during infancy in CHH boys with congenital cryptorchidism.

**Table 3:** Summary of the studies on hormonal treatment during infancy in boys with congenital hypogonadotropic hypogonadism and cryptorchidism.

**First authors**	**Study design** **Country of investigation ** **Study subjects (n)**	**Treatment**	**Age at hormone measurements**	**Testicular location after treatment**	**Hormone results**
Kohva et al. (242)	Retrospective cohort study Finland Study reported both short- and long-term data *Short -term follow-up data* five CHH boys (two boys with congenital bilateral cryptorchidism and three boys with acquired cryptorchidism). All five boys underwent bilateral orchiopexy at the age of 2 ± 0.7 y	rhFSH (for 3–4.5 months) and testosterone enanthate (for 3 months) Treatment started at the age of 2.5 ± 1.3 months	Short term data of five CHH boys: serum inhibin B before (range, 3 days to 2.1 months before), during and after (range, 0.1–1.4 months after) treatment with rhFSH plus testosterone	(All boys underwent bilateral orchiopexy.)	Inhibin B levels increased after starting treatment with rhFSH and testosterone, and the levels lowered after discontinuation of treatment.
	*Long-term comparison data (during adolescence)* Three of five CHH boys with a history of cryptorchidism who received rhFSH and testosterone in infancy had available data at adolescence (age, 10.0–12.8 y) vs. retrospective controls (8 CHH boys, aged 12.7–17.8 y) without previous gonadotropin treatment (six boys had cryptorchidism.)		data at adolescence of three CHH boys with a history of cryptorchidism treated with rhFSH and testosterone during infancy vs. six untreated CHH boys with history of cryptorchidism		*CHH boys treated (n = 3) vs. not treated with rhFSH and testosterone (n = 6)* *(compared during adolescence)* Inhibin B levels of the CHH boys with a history of cryptorchidism who received (*n =* 3) and did not receive treatment (*n =* 6) during infancy were not different.
Lambert and Bougneres (245)	Intervention study USA Eight CHH boys with cryptorchidism (five abdominal testes and three high scrotal testes), aged 0.25–11 months	Subcutaneous infusion at a daily rate of rhLH 50 IU and rhFSH 75–150 IU for 6 ± 0.58 months	Age (mean ± SD): 6.0 ± 3.8 months (range: 0.25–11 months)	Six boys—complete testicular descent Two boys—partial testicular descent One boy—testis reascended Testes gained normal size.	FSH, LH, and testosterone increased to normal range.
Papadimitriou et al. (244)	Intervention study Greece Ten boys with bilateral cryptorchidism in intra-abdominal or inguinal position and micropenis with the absence of mini-puberty Follow-up for 3–10 years	Daily subcutaneous injections of Pergoveris (LH/FSH 75/150 IU) for 3 months started from median age of 0.35 (0.19–0.78) year	Twenty-four hours after the last injection of LH/FSH	All testes descended to the scrotal position within 3 months after treatment. After 3–10 years of follow-up, all testes were still in scrotal position.	*24 hours after last injection of LH/FSH* LH, FSH, inhibin B, and T levels increased to normal or supranormal levels.

*CHH, congenital hypogonadotropic hypogonadism; r-hFSH, recombinant human FSH; rhLH, recombinant human LH*.

Overall, the data showed that gonadotropin administration during mini-puberty is relatively effective in inducing testicular descent in CHH patients. However, the available data are very limited and all of the studies are small due to the rarity of this condition. The European consensus statement on congenital hypogonadotropic hypogonadism published in 2015 recommends orchiopexy before the age of 1 year as the standard treatment for cryptorchidism (123), which is based mostly on the evidence from boys with isolated cryptorchidism, not in a specific subgroup of CHH boys (225).

## Unanswered Questions and Future Directions

The cause of isolated cryptorchidism is still not fully understood, and therefore more studies investigating the mechanisms of cryptorchidism are still needed. The studies on hormonal treatment during mini-puberty in CHH boys showed promising results on testicular descent, however, the treatment outcomes were observed without randomization of the subjects. Additionally, long-term outcomes are still largely unknown. Therefore, randomized, multi-center studies that explore the role of gonadotropin or GnRH treatment in mini-puberty and peri-puberty in CHH patients with cryptorchidism are necessary to gain more knowledge on the effects of this treatment.

## Conclusion

Hypogonadism can be a cause or effect of cryptorchidism. Conditions associated with decreased androgen levels or actions may present with unilateral or bilateral cryptorchidism. On the other hand, hypogonadism can be sequelae from cryptorchidism. Several studies have suggested that cryptorchidism, particularly bilateral cryptorchidism, is associated with reduced spermatogenesis and inhibin B levels and increased FSH levels in adulthood. Similar hormonal findings have been observed during mini-puberty and puberty in some studies. Several studies have suggested reduced Leydig cell function during mini-puberty, at least in more severe forms of cryptorchidism. More studies are needed on the association between later testicular function, especially endocrine function, and operation age or type of cryptorchidism. Furthermore, studies exploring the underlying mechanisms of cryptorchidism that cause reduced testicular hormone production are needed. Lastly, more studies investigating predictors of hypogonadism in adulthood and interventions to reduce the risk of hypogonadism are necessary.

## Author Contributions

All the authors have contributed to writing of the review by providing texts and commenting the whole paper. They all take responsibility of the final manuscript.

### Conflict of Interest

The authors declare that the research was conducted in the absence of any commercial or financial relationships that could be construed as a potential conflict of interest.
